# Exercise Intervention Promotes the Growth of Synapses and Regulates Neuroplasticity in Rats With Ischemic Stroke Through Exosomes

**DOI:** 10.3389/fneur.2021.752595

**Published:** 2021-10-28

**Authors:** Chen Li, Changkai Ke, Yue Su, Chunxiao Wan

**Affiliations:** Department of Physical Medicine and Rehabilitation, Tianjin Medical University General Hospital, Tianjin, China

**Keywords:** MCAO, exercise intervention, exosomes, neuroplasticity, synapses

## Abstract

**Background:** Stroke is the leading cause of death and disability. Exercise produces neuroprotection by improving neuroplasticity. Exercise can induce exosome production. According to several studies, exosomes are involved in repairing brain function, but the relationship and mechanism of exercise, exosomes, and neuroprotection have not been elucidated. This study intends to explore the relationship and potential mechanism by observing the changes in the exosome level, infarct volume, neurological function and behavioral scores, synapses, and corticospinal tract (CST).

**Methods:** Rats were randomly divided into four groups: a sham operation (SHAM) group, middle cerebral artery occlusion (MCAO) with sedentary intervention (SED-MCAO) group, MCAO with exercise intervention (EX-MCAO) group, and MCAO with exercise intervention and exosome injection (EX-MCAO-EXO) group. The exercise intervention was started 1 day after MCAO and lasted for 4 weeks. All rats were assessed using the modified neurological severity score (mNSS). The levels of exosomes in serum and brain, gait analysis, and magnetic resonance scan were performed 1 and 4 weeks after the intervention. After 4 weeks of intervention, the number of synapses, synaptophysin (Syn), and postsynaptic density protein 95(PSD-95) expression was detected.

**Results:** After 4 weeks of intervention, (1) the EX-MCAO and EX-MCAO-EXO groups showed higher serum exosome (*p*_EX−MCAO_ = 0.000, *p*_EX−MCAO−EXO_ = 0.000) and brain exosome (*p*_EX−MCAO_ = 0.001, *p*_EX−MCAO−EXO_ = 0.000) levels than the SED-MCAO group, of which the EX-MCAO group had the highest serum exosome (*p* = 0.000) and the EX-MCAO-EXO group had the highest brain exosome (*p* = 0.03) levels. (2) The number of synapses in the EX-MCAO (*p* = 0.032) and EX-MCAO-EXO groups (*p* = 0.000) was significantly higher than that in the SED-MCAO group. The EX-MCAO-EXO group exhibited a greater number of synapses than the EX-MCAO (*p* = 0.000) group. (3) The synaptic plasticity-associated proteins were expressed significantly higher in the EX-MCAO (*p*_Syn_ = 0.010, *p*_PSD−95_ = 0.044) and EX-MCAO-EXO (*p*_Syn_ = 0.000, *p*_PSD−95_ = 0.000) groups than in the SED-MCAO group, and the EX-MCAO-EXO group (*p*_Syn_ = 0.000, *p*_PSD−95_ = 0.046) had the highest expression. (4) Compared with the SED-MCAO group, the EX-MCAO group had significantly improved infarct volume ratio (*p* = 0.000), rFA value (*p* = 0.000), and rADC (*p* = 0.000). Compared with the EX-MCAO group, the EX-MCAO-EXO group had a significantly improved infarct volume ratio (*p* = 0.000), rFA value (*p* = 0.000), and rADC value (*p* = 0.001). (5) Compared with the SED-MCAO group, the EX-MCAO group (*p* = 0.001) and EX-MCAO-EXO group (*p* = 0.000) had significantly lower mNSS scores and improved gait. (6) The brain exosome levels were negatively correlated with the mNSS score, infarct volume ratio, and rADC value and positively correlated with the rFA value, Syn, and PSD-95 expression. The serum and brain exosome levels showed a positive correlation.

**Conclusions:** Exercise intervention increases the serum exosome level in MCAO rats, which are recruited into the brain, leading to improved synaptic growth and CST integrity, a reduced infarct volume, and improved neurological function and gait.

## Introduction

Stroke is the leading cause of death and disability worldwide. Approximately 15 million people worldwide suffer from stroke each year, and ~5 million people die from stroke (1–3). Cerebral ischemia (CI) accounts for 87% of cases, results in movement disorders, and seriously affects quality of life (4). With the development of science and technology, new technologies and treatments have emerged, but the best method is still being explored.

Some studies have confirmed that exercise after cerebral ischemic injury exerts a neuroprotective effect by promoting angiogenesis, neurogenesis, and neuroplasticity (5), and improves functional recovery after stroke (6). Recently, some studies have found that the beneficial effects of exercise may be related to the regulation of exosome levels (7). Studies have found that exercise promotes the release of exosomes. Exosomes play an important role in cell communication (8–10) and have independent potential in the treatment of various diseases, such as the immune response, cardiovascular disease, and nervous system disease (10, 11). A number of studies have confirmed that exosomes cross the blood-brain barrier (BBB) through fluorescent labeling (12, 13). Wang et al. found that moderate exercise increases circulating levels of endothelial progenitor cell-derived exosomes. These circulating exosomes are involved in brain tissue repair, including reducing cell apoptosis, the cerebral infarct volume, and angiogenesis disorders (14, 15).

Exosomes are membrane-bound vesicles with a diameter of 30–150 nm (16) that carry proteins, lipids, and genetic material (3). Exosomes released by brain cells carry more than 2,000 proteins involved in brain repair functions (17), including synaptic transmission, neural projection, and synaptic growth (18). There are many miRNAs related to the neuroprotection of exosomes, such as miR-17-92, miR-126, and miR-124, which also play a key role in neurological recovery after stroke (19). Exosomes induce long-term brain protection, promote nerve recovery (17), and provide a new treatment for neuroprotection and repair after stroke (20).

Although exosomes have therapeutic potential in many diseases (21), few studies have directly confirmed that exercise after CI increases exosome levels and protects the brain. This study focuses on the effect, relationship, and mechanism of exercise on exosomes and neuroplasticity after CI.

The neurons and conduction bundles are damaged after CI and result in an increased infarct volume, motor dysfunction, and behavioral disorders. Magnetic resonance imaging (MRI) is used to assess brain structure *in vivo* (22). Diffusion tensor imaging (DTI) and diffusion weighted imaging (DWI) technology quantify the remodeling of white matter and quantitatively predict the degree of restriction of movement (23, 24), motor function, and neuroplasticity (25, 26). Diffusion tensor tractography (DTT) technology can be used to reconstruct the corticospinal tract (CST).

Synapses are the basic unit of brain function (27). Synaptophysin (Syn) and postsynaptic density protein 95 (PSD-95) are an important marker of synaptic plasticity in the brain. Syn is located in presynaptic vesicles and is closely related to the structure and function of synapses (28). PSD-95 is extensively studied in synaptic plasticity (29). After stroke, the synaptic structure is disintegrated, the axon ends are degenerated and disappear, the Syn and PSD-95 expression is reduced (30). With the enhancement of neuroplasticity, the synaptic structure begins to be repaired, and the number of synapses and synaptic plasticity-associated protein expression gradually increases (31).

In this study, we performed exercise intervention in rats after cerebral artery occlusion (MCAO). We observed the effects of exercise on the serum and brain exosome levels, synapses, CST, infarct volume, motor function, and gait parameters. We further injected exosomes *via* the tail vein to clarify the effect of exosomes. We analyzed the relationships not only between brain exosomes and the mNSS score, infarct volume, rFA value, rADC value, Syn, and PSD-95 expression but also between serum and brain exosome levels.

This study aims to explore the mechanism of exercise intervention to improve the neuroplasticity of MCAO rats, with the goal of providing new treatment strategies for clinical patients with stroke, enhancing the rehabilitation effect, and improving the quality of life.

## Materials and Methods

### Experimental Animals and Groups

**Figure 1:**
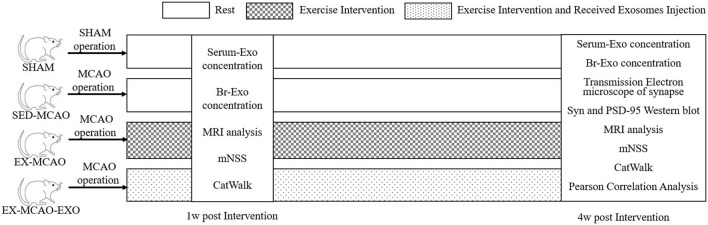
Schematic of the experimental protocol.

Sixty adult male Sprague-Dawley rats (8–10 weeks old, weighing 280–320 g, Beijing Huafukang Biotechnology Co., Ltd., China) were used in this study. They were randomly divided into four groups: sham operation (SHAM) group, MCAO with sedentary intervention (SED-MCAO) group, MCAO with exercise intervention (EX-MCAO) group, and MCAO with exercise intervention and received exosome injection (EX-MCAO-EXO) group. There were 15 rats in each group, and 6 of them were randomly sacrificed after 1 week of intervention to detect the level of exosomes in serum and brain. The protocol schematic is shown in Figure 1.

The experimental procedure was performed according to the National Institutes of Health (NIH) Laboratory Care and Use Guidelines to minimize the number of animals used and the pain of animals during the experiment and was approved by the Ethics Committee of Tianjin Medical University (TMUaMEC 2018037).

### The MCAO Model

The modified Longa thread embolization method was used to prepare the MCAO model (32). Under anesthesia (sodium pentobarbital, 40 mg/kg, i.p.), the rats were fixed in supine position and opened along the median carotid line. Subcutaneous tissue and anterior carotid muscles were passively separated, the left common carotid artery and internal and external carotid artery were separated, and a fine silicon-coated surgical nylon monofilament (2838-A4, 0.38 ± 0.02 mm, Beijing Xinong Technology Co., Ltd., China) was inserted into the left internal carotid artery to block the middle cerebral artery (up to ~18.0–20.0 mm distal to the bifurcation of the carotid artery) for 60 min and then removed for reperfusion. After the rats awakened, a Longa score of 1–3 points was used as the standard for successful modeling (33).

### Intervention Method

#### Exercise Intervention

According to our previous research (34), all rats underwent a preliminary intervention on the treadmill for 3 days of adaptation and then were randomly divided into four groups. The intervention began 1 day after MCAO and lasted for 4 weeks. Exercise was conducted on a treadmill (ZS-PT of Zhongshi Dichuang Company, Beijing, China, angle of 0°, speed of 12 m/min) for 30 min daily, 5 days/week. The sedentary intervention was conducted on a motionless treadmill for 30 min daily, 5 days/week.

#### Exercise Intervention and Exosome Injection

The brain-derived exosomes were taken from the brain tissue of healthy rats, and the protein concentration was quantified with a BCA kit (Solarbio, Beijing, China). At 24 h post MCAO, rats in the EX-MCAO-EXO group received brain-derived exosomes containing 100 μg of protein in 0.5 ml phosphate-buffered saline (PBS) and were slowly injected *via* the tail vein (35–37). The exercise intervention was the same as the EX-MCAO group.

### Isolation, Characterization, and Labeling of Exosomes

#### Exosome Isolation

After 1 and 4 weeks of intervention, the serum exosomes and brain-derived exosomes were isolated as follows.

Isolation of serum exosomes: Blood samples were incubated for 30 min at room temperature to induce clotting and then centrifuged for 20 min at 1,500 rpm. Serum samples were centrifuged at 1,500× g for 20 min and 13,000× g for 3 min to thoroughly remove cellular debris. Next, supernatants were filtered through a 0.22 μm pore size filter to exclude particles >220 nm in diameter. Ultracentrifugation was performed for 2 h at 100,000× g, and the precipitates were resuspended in PBS. All centrifugation steps were performed at 4°C (38).

Isolation of brain-derived exosomes: Brain tissue was ground into a homogenate. The extraction method was the same as that used for serum exosomes.

#### Exosome Characterization

Nanoparticle tracking analysis (NTA, Nanosight NS300, UK) and transmission electron microscopy (TEM, Hitachi HT7700, Japan) were used to observe the size and morphology of exosomes, and Western blotting was used to identify the exosome surface marker proteins CD9 and TSG101 (39).

#### Exosome Labeling

Purified exosomes containing 100 μg of protein were incubated with DIR (Umibio, Shanghai, China) for 30 min at 37°C. Unbound dye was removed by centrifugation at 100,000× g for 2 h, and then the pellet was washed with precooled PBS. After 24 h of injection of exosomes into the tail vein, the IVIS Spectrum system (Caliper, USA) was used for exosome labeling.

### Neurological Function Assessment and Gait Analysis

The modified neurological severity score (mNSS) was used to evaluate neurological function (40). The total score ranges from 0 to 18 points, and a higher score indicates a more severe neurological deficit. Scoring was performed 1, 3, 7, 14, 21, and 28 days after MCAO.

One and 4 weeks after the exercise intervention, the gait was determined (CatWalk Analysis System, Noldus Information Technology, Wageningen, The Netherlands) to evaluate gait restoration. Each evaluation was repeated at least three times, and gait was assessed as the animal that walked along the set length range of the glass plate within a specified time (within 10 s) at each time point as the system described. The whole experimental process was completed in a dark and silent environment, according to the instructions provided with the device. It generated gait parameters, such as pace, paw print area, and gait mode.

### MRI Scan

MRI was performed 1 and 4 weeks after the intervention using a 3.0 T MRI scanner (MagnetomVerio, SIEMENS, Germany). T2-weighted imaging (T2WI) was conducted to detect the infarct volume from bregma +1.7 to−6.3 mm using a T2-SPACE sequence with the following parameters: TR = 1,000 ms, TE = 155 ms, FA = 120°, FOV = 60 × 60 mm^2^, matrix = 192 × 192, and 72 slices of 0.4 mm thickness without a gap. Cerebral infarct volume was quantified as previously described (41), summing all the lesion areas measured in all slices and multiplying by the slice thickness.

Using DTI and DWI techniques, the same level (bregma-1.08 mm -4.20 mm) was selected to measure the fraction anisotropy (FA) and apparent diffusion coefficient (ADC) in the area of the injured inner capsule and the corresponding inner capsule area on the contralateral side. DTT trace processing was performed using the DTI image. rFA = affected side FA/unaffected side FA. rADC = ADC on the affected side/ADC on the unaffected side.

### Western Blot Analysis

Brain tissues from the peri-infarcted region were dissected after 4 weeks of intervention. After the protein concentration was quantified with a BCA kit (Solarbio, Beijing, China), the total protein was separated and transferred to polyvinylidene difluoride membranes (Millipore, USA); the membrane was then blocked. Anti-Syn (Abcam, UK, 1:2,000), anti-PSD95 (Affinity, China, 1:1,000) and β-actin (Cell Signaling, USA, 1:1,000) antibodies were incubated with the membrane overnight at 4°C. After washing, the membrane was incubated with horseradish peroxidase-conjugated secondary antibodies (Cell Signaling, USA, 1:2,000) for 1 h at room temperature. ImageJ software was used to detect the gray value of the band, and the protein level was quantitatively analyzed.

The membrane was incubated with primary antibodies against CD9 (Abcam, UK, 1:2,000) and TSG101 (Abcam, UK, 1:2,000) overnight at 4°C to detect exosomal proteins, and the other methods were the same as described above.

### Transmission Electron Microscopy (TEM)

Brain tissues (1 mm^3^) from the peri-infarcted region were fixed with 6% glutaraldehyde at 4°C. Tissues were sliced by vibratome and stained with lead. At least 10 sections per rat were selected and photographed using a TEM (Hitachi HT7700, Japan). Synapses were measured using Image-Pro Plus 6.0 software (Media Cybernetics, USA).

### Statistical Analysis

Statistical software SPSS 25.0 (SPSS Inc., Armonk, NY, USA) and GraphPad Prism 6.0 (GraphPad Software, Inc., La Jolla, CA, USA) were used. Data are presented as the means ± standard deviations (SD). The Shapiro-Wilks test was used to verify the normal distribution of the data. One-way analysis of variance (ANOVA) followed by LSD-t *post hoc* test was used to assess the differences of synapse numbers, synaptic plasticity-associated protein expression, and gait parameters. Repeated measures ANOVA determined differences among groups with time, such as the levels of serum exosomes and brain tissue exosomes, MRI parameters, and mNSS score. *p* < 0.05 was considered significant. Pearson's correlation analysis was used to determine correlations, and *p* < 0.05 indicated that the difference was significant.

## Results

### Exosome Characterization and Labeling

**Figure 2:**
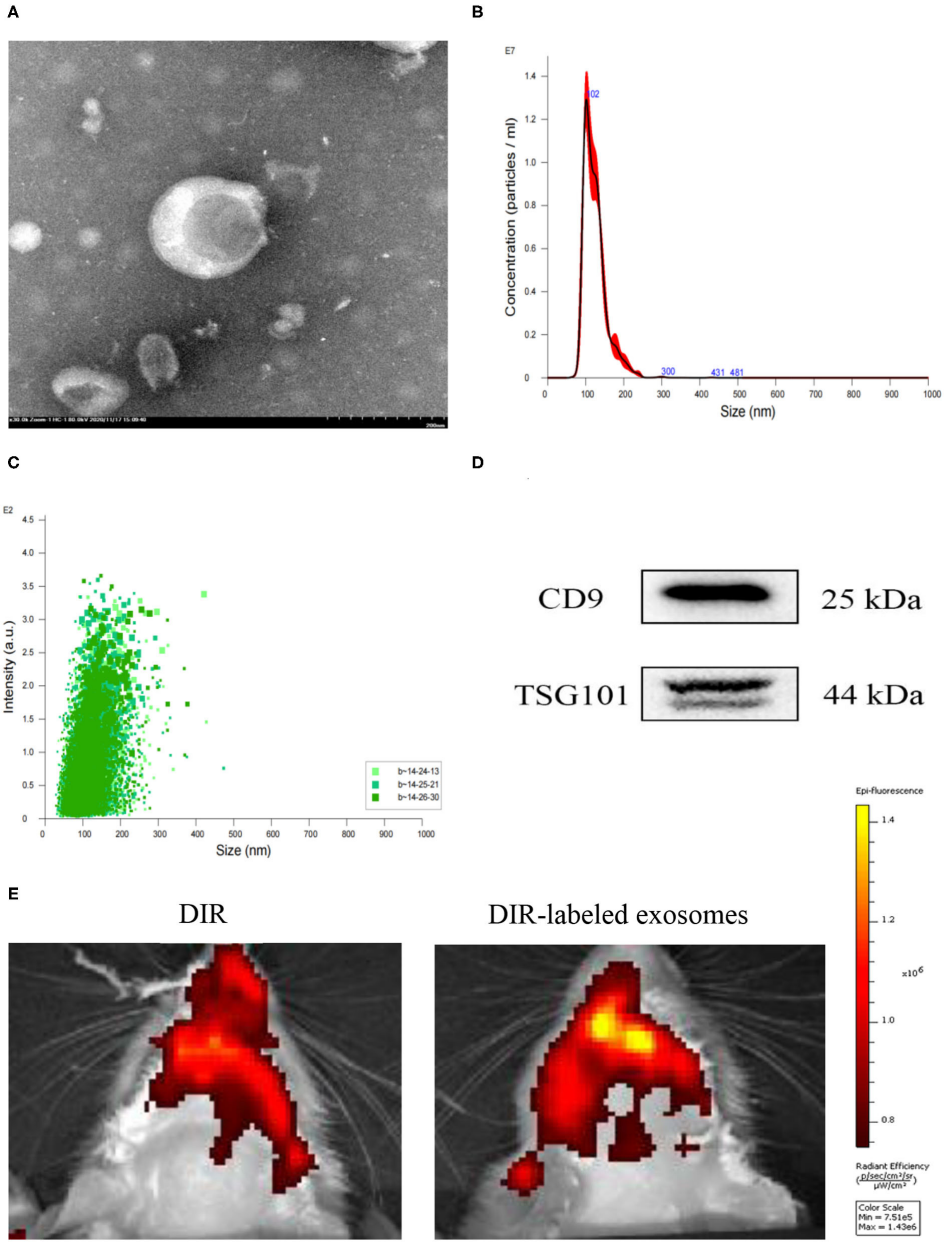
Identification and labeling of exosomes. **(A)** Morphological observation of exosomes under a transmission electron microscope (TEM, scale bar = 200 nm). **(B,C)** Particle size distribution measured using NTA. **(D)** The exosome surface marker proteins CD9 and TSG101 were detected using Western blotting. **(E)** Distribution of DIR-labeled exosomes injected through the tail vein in the brain.

The morphology, particle size, and surface marker proteins were identified. The identity of exosomes was confirmed using TEM, which revealed a classic “cup-shaped” structure (Figure 2A). The particle size distribution of exosomes was analyzed using NTA, and the average particle size was 124.4 ± 0.4 nm (Figures 2B,C). Western blot analysis detected the surface marker proteins CD9 and TSG101, which were consistent with the biological characteristics of exosomes (Figure 2D).

One day after MCAO, 100 μg protein exosomes were injected through the tail vein. DIR biodistribution detection was performed after 24 h. Compared with DIR dye injection, DIR-labeled exosomes rats exhibited a higher signal in the brain (Figure 2E).

### The Levels of Exosomes in the Serum and Brain of MCAO Rats at 1 and 4 Weeks of the Exercise Intervention

The levels of serum exosomes and brain tissue exosomes at 1 and 4 weeks were evaluated by repeated measures analysis of variance. The results showed the following.

**Figure 3:**
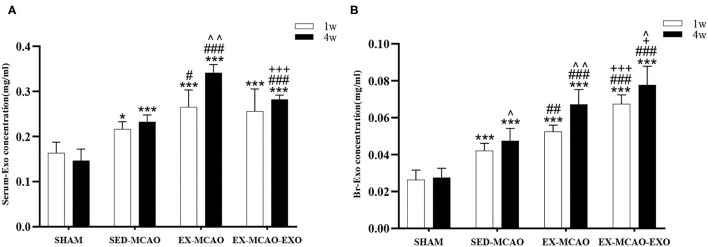
Concentrations of exosomes in the serum and brain of MCAO rats after 1 and 4 weeks of intervention. **(A)** Serum exosome concentrations. **(B)** Brain exosome concentrations. Serum-Exo concentration: serum exosome concentration, Br-Exo concentration: brain exosome concentration. ^∧^*p* < 0.05 and ^∧∧^*p* < 0.05 compared with the corresponding levels at 1 week within groups. *p < 0.05 and ***p < 0.001 compared with the corresponding SHAM group. ^#^*p* < 0.05, ^##^*p* < 0.01, and ^###^*p* < 0.001 compared with the corresponding SED-MCAO group. ^+^*p* < 0.05 and ^+++^*p* < 0.001 compared with the corresponding EX-MCAO group. Means ± standard deviations, *n* = 6/group.

Comparison of serum exosome levels (Figure 3A) time factor [*F*_time (1,20)_ = 13.494, *p*_time_ = 0.002] and intervention methods [*F*_intervention__(3,20)_ = 52.722, *p*_intervention_ = 0.001] had a significant effect on serum exosome levels. There was an interaction between time and intervention [*F*_time×*intervention* (3,20)_ = 7.855, *p* = 0.001]. Subsequent multiple comparisons showed that compared with the SHAM group, the serum exosome level in the MCAO group was increased (*p* < 0.05). After 1 week of intervention, the EX-MCAO group (*p* = 0.022) exhibited significantly higher serum exosome levels than the SED-MCAO group. After 4 weeks of intervention, the levels in the EX-MCAO group (*p* = 0.000) and EX-MCAO-EXO group (*p* = 0.000) were significantly higher than those in the SED-MCAO group, among which the EX-MCAO group (*p* = 0.000) had the highest serum exosome level. A simple effect analysis of time showed that the serum exosome level in the EX-MCAO group was significantly increased at 4 weeks (*p* = 0.001).

Comparison of brain exosome levels (Figure 3B) time and intervention method interaction at the level of brain exosomes [*F*_time×*intervention*__(3,20)_ = 5.901, *p* = 0.005], and their main effects were also meaningful [*F*_time__(1,20)_ = 41.750, *p*_time_ = 0.000; *F*_intervention__(3,20)_ = 73.761, *p*_intervention_ = 0.000]. Subsequent multiple comparisons showed that after intervention, the brain exosome levels in the MCAO groups (*p* = 0.000) were higher than those in the SHAM group. The EX-MCAO (*p*_1w_ = 0.001, *p*_4w_ = 0.001) and EX-MCAO-EXO groups (*p*_1w_ = 0.000, *p*_4w_ = 0.000) had significantly higher brain exosome levels than the SED-MCAO group, of which the EX-MCAO-EXO group had the highest level (*p*_1w_ = 0.000, *p*_4w_ = 0.03). A simple effect analysis of time showed that the level of brain exosomes in the SHAM group showed no difference between 1 and 4 weeks (*p*_SHAM_ = 0.690), while the others were significantly improved (*p*_SED−MCAO_ = 0.045, *p*_EX−MCAO_ = 0.001, *p*_EX−MCAO−EXO_ = 0.015).

### Synapse Detection Using TEM

**Figure 4:**
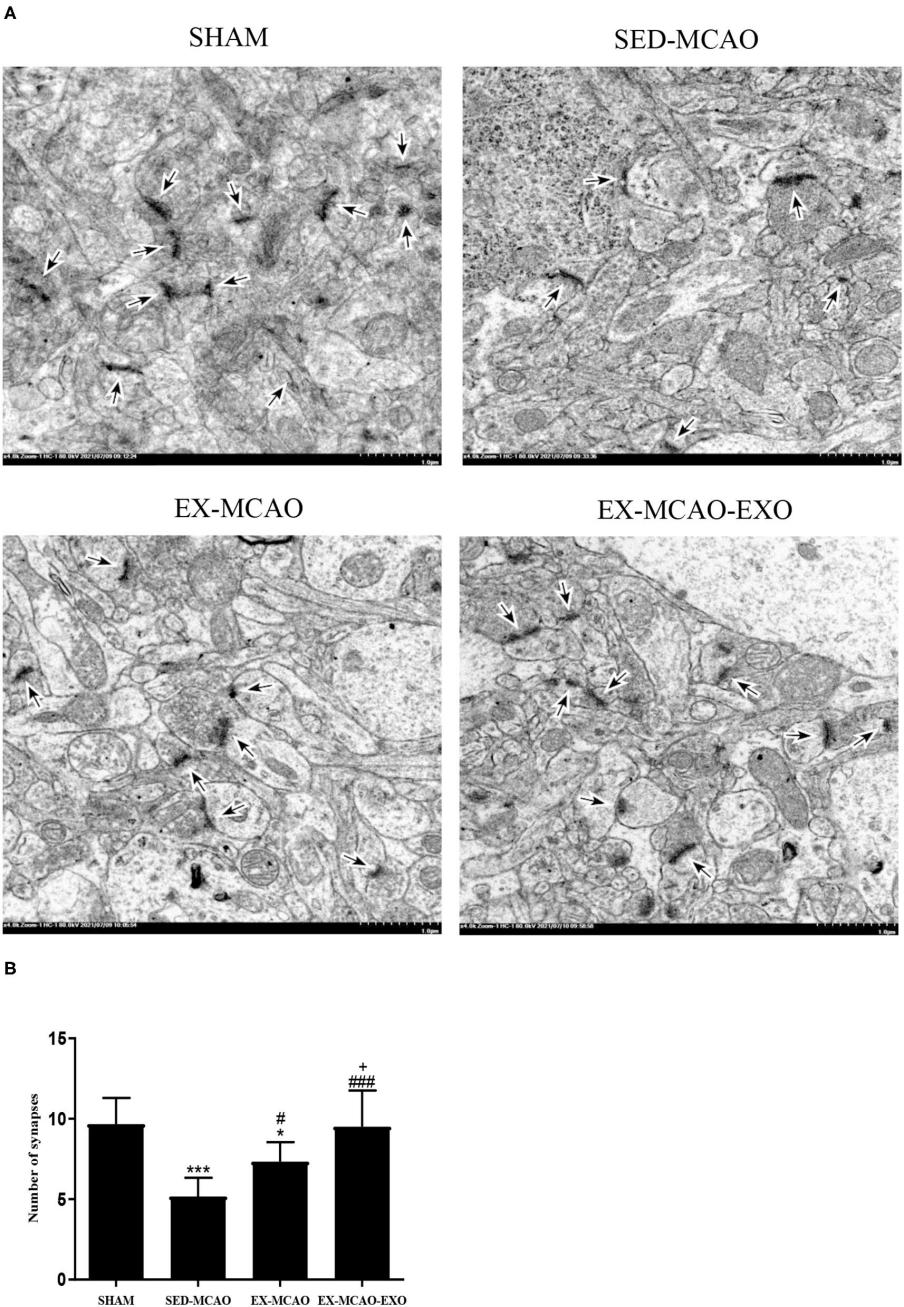
Synapses were detected using transmission electron microscopy. **(A)** Representative images of synapses in the four groups. The synapse was observed under an electron microscope at ×4.0k magnification (bar = 1.0 μm). **(B)** The number of synapses. *p < 0.05 and ***p < 0.001 compared with the corresponding SHAM group. ^#^*p* < 0.05 and ^###^*p* < 0.001 compared with the corresponding SED-MCAO group. ^+^*p* < 0.05 compared with the corresponding EX-MCAO group. Means ± standard deviations, *n* = 3/group.

The synapse was detected using TEM in a ×4.0k field of view (Figure 4). After 4 weeks of intervention, the number of synapses in the SED-MCAO (*p* = 0.000) and EX-MCAO groups (*p* = 0.022) was significantly decreased compared with the SHAM group. A significant difference was not observed between the EX-MCAO-EXO and SHAM groups (*p* = 0.861). The number of synapses in the EX-MCAO (*p* = 0.032) and EX-MCAO-EXO (*p* = 0.000) groups was significantly higher than that in the SED-MCAO group, among which the EX-MCAO-EXO group (*p* = 0.000) had the highest synapse numbers.

### Expression of the Syn and PSD-95 in the Peri-Infarct Region of the Brain

**Figure 5:**
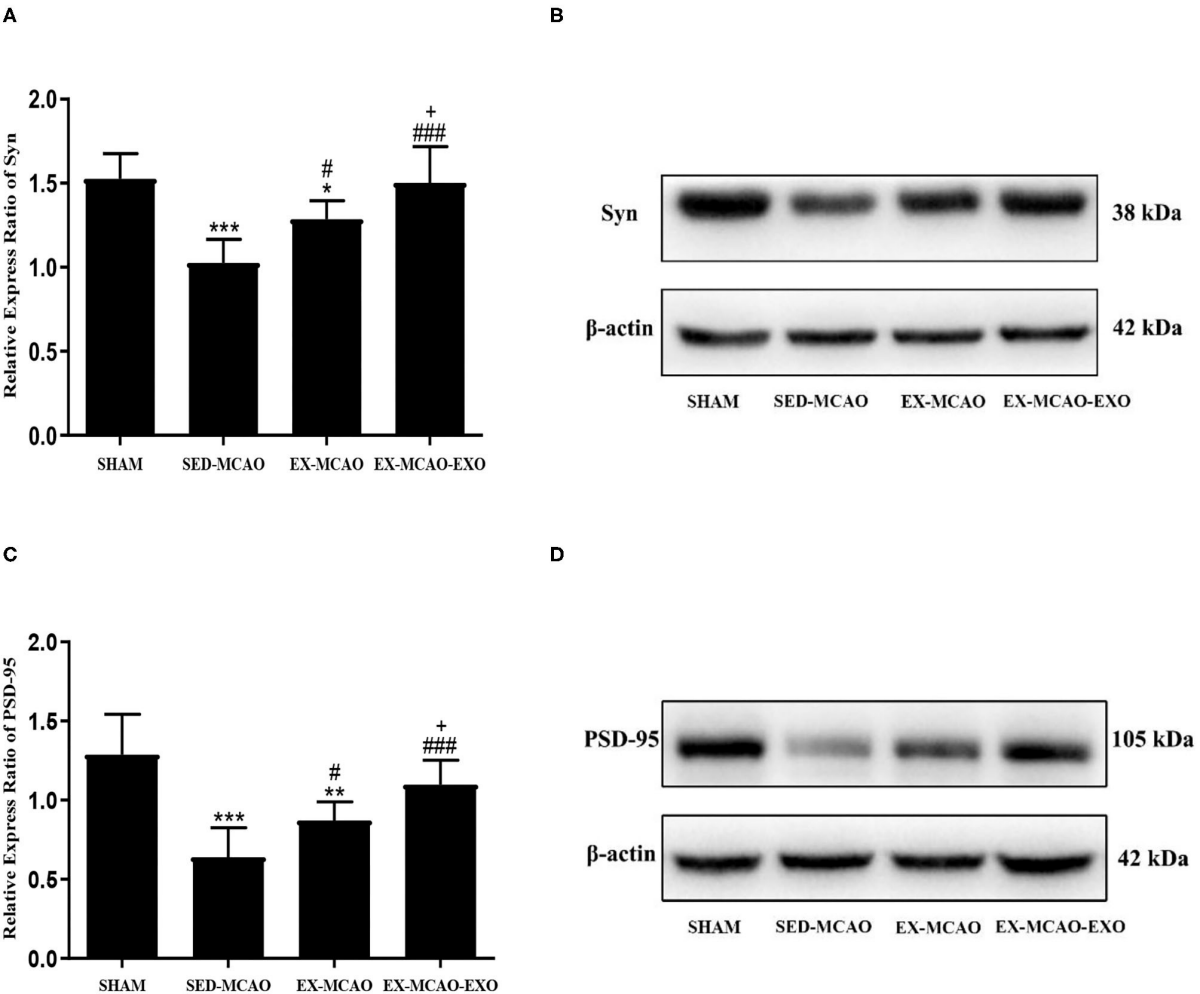
The relative level of the synaptic plasticity-associated protein expression in the peri-infarcted region of the brain. **(A)** Relative level of the Syn protein. **(B)** Western blotting was used to detect Syn expression. **(C)** Relative level of the PSD-95 protein. **(D)** Western blotting was used to detect PSD-95 expression. *p < 0.05, **p < 0.01, and ***p < 0.001 compared with the corresponding SHAM group. ^#^*p* < 0.05 and ^###^*p* < 0.001 compared with the corresponding SED-MCAO group. ^+^*p* < 0.05 compared with the corresponding EX-MCAO group. Means ± standard deviations, *n* = 6/group.

After 4 weeks of intervention (Figure 5), the level of synaptic plasticity-associated proteins in the SED-MCAO (*p*_Syn_ = 0.000, *p*_PSD−95_ = 0.000) and EX-MCAO (*p*_Syn_ = 0.016, *p*_PSD−95_ = 0.001) groups was significantly reduced compared with the SHAM group. A significant difference was not observed between the EX-MCAO-EXO and SHAM groups (*p*_Syn_ = 0.803, *p*_PSD−95_ = 0.093). The synaptic plasticity-associated proteins were expressed significantly higher in the EX-MCAO (*p*_Syn_ = 0.010, *p*_PSD−95_ = 0.044) and EX-MCAO-EXO (*p*_Syn_ = 0.000, *p*_PSD−95_ = 0.000) groups than in the SED-MCAO group, and the EX-MCAO-EXO group (*p*_Syn_ = 0.000, *p*_PSD−95_ = 0.046) had the highest expression.

### MRI Parameters

**Figure 6:**
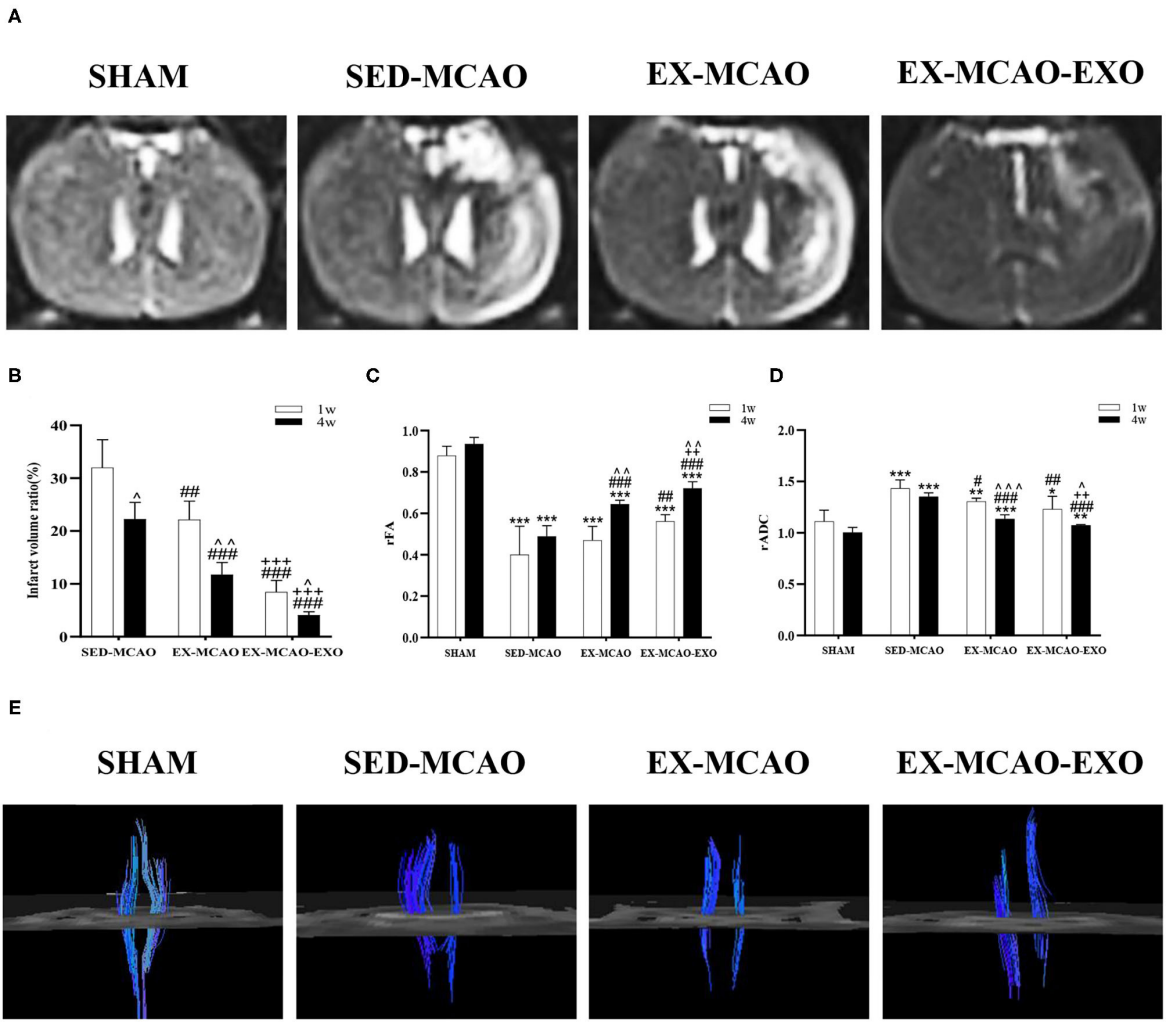
Magnetic resonance imaging of rats after the exercise intervention. **(A)** Brain transverse spin-spin relaxation time of Mxy weight image (T2WI) sequence images after 4 weeks of intervention. **(B)** Infarct volume ratio. **(C)** rFA value after cerebral infarction. **(D)** rADC value after cerebral infarction. **(E)** Corticospinal tract tracking diagram after 4 weeks of intervention. ^∧^*p* < 0.05, ^∧∧^*p* < 0.01, and ^∧∧∧^*p* < 0.001 compared with the corresponding parameter at 1 week within groups. *p < 0.01, **p < 0.05, and ***p < 0.001 compared with the corresponding SHAM group. ^#^*p* < 0.05, ^##^*p* < 0.01, and ^###^*p* < 0.001 compared with the corresponding SED-MCAO group. ^++^*p* < 0.01 and ^+++^*p* < 0.001 compared with the corresponding EX-MCAO group. Means ± standard deviations, *n* = 6/group.

After 1 and 4 weeks of intervention, each group was scanned by MRI (Figures 6A,B). The influence of time and intervention method on infarct volume ratio were statistically significant [*F*_time__(1,15)_ = 53.601, *p*_time_ = 0.000; *F*_intervention (2,15)_ = 144.328, *p*_intervention_ = 0.000], but there was no interaction between time and intervention methods [*F*_time×*intervention* (2, 15)_ = 2.930, *p* = 0.084]. After intervention, the infarct volume ratio in the EX-MCAO (*p*_1w_ = 0.001, *p*_4w_ = 0.000) and EX-MCAO-EXO (*p*_1w_ = 0.000, *p*_4w_ = 0.000) groups was significantly reduced compared with that in the SED-MCAO group. The infarct volume ratio in the EX-MCAO-EXO group (*p*_1w_ = 0.000, *p*_4w_ = 0.000) was lower than that in the EX-MCAO group. A simple effect analysis of time showed that infarct volume ratio in the three groups decreased over time (*p*_SED−MCAO_ = 0.015, *p*_EX−MCAO_ = 0.002, *p*_EX−MCAO−EXO_ = 0.010).

The FA and ADC values in the area of the injured inner capsule and the corresponding inner capsule area on the contralateral side were measured.

Time and intervention could affect the level of rFA [*F*_time (1,20)_ = 44.758, *p*_time_ = 0.000; *F*_intervention (3,20)_ = 113.683, *p*_intervention_ = 0.000], but there was no interactive effect between both factors [*F*_time×*intervention* (3,20)_ = 2.474, *p* = 0.091]. The rFA values in the MCAO groups (*p* = 0.000) were lower than those in the SHAM group after 1 and 4 weeks of intervention (Figure 6C). After 1 week of intervention, compared with that in the SED-MCAO group, the rFA value in the EX-MCAO-EXO group (*p* = 0.003) was significantly increased. After 4 weeks of intervention, the rFA values in the EX-MCAO (*p* = 0.000) and EX-MCAO-EXO (*p* = 0.000) groups were significantly higher than that in the SED-MCAO group, among which the EX-MCAO-EXO group (*p* = 0.001) had the highest rFA value. The comparison of 4 and 1 week of interventions showed that the rFA values in the EX-MCAO and EX-MCAO-EXO group increased significantly (*p*_EX−MCAO_ = 0.004, *p*_EX−MCAO−EXO_ = 0.001), while that in the SHAM and SED-MCAO group had no differences.

Time and intervention in rADC values did not interact with each other [*F*_time×*intervention* (3,20)_ = 1.091, *p* = 0.367]. It was statistically significant that time and intervention make effect on the value of rADC [*F*_time (1,20)_ = 40.294, *p*_time_ = 0.000; *F*_intervention (3,20)_ = 44.456, *p*_intervention_ = 0.000]. The rADC values in the MCAO groups (*p* < 0.05) were higher than that in the SHAM group (Figure 6D). The rADC values in the EX-MCAO (*p*_1w_ = 0.030, *p*_4w_ = 0.000) and EX-MCAO-EXO (*p*_1w_ = 0.001, *p*_4w_ = 0.000) groups were significantly lower than that in the SED-MACO group. After 4 weeks of intervention, the rADC in the EX-MCAO-EXO group (*p* = 0.006) was significantly lower than that in the EX-MCAO group. In the comparison of intervention between two points, the rADC was significantly reduced in EX-MCAO and EX-MCAO-EXO group (*p*_EX−MCAO_ = 0.000, *p*_EX−MCAO−EXO_ = 0.022) rather than in SHAM and SED-MCAO group (*p*_SED−MCAO_ = 0.069, *p*_SHAM_ = 0.112).

After 4 weeks of intervention, the DTI images were traced (Figure 6E). The bilateral CST of the SHAM group was symmetrical and large, with good continuity. Compared with the SHAM group, the CSTs in the SED-MCAO group, EX-MCAO group, and EX-MCAO-EXO group were asymmetric, and the affected sides were significantly slimmer. In the SED-MCAO group, the affected CST was significantly less than the unaffected side, and some fiber bundles were interrupted in the affected CST. Compared with the SED-MCAO group, the affected CST in the EX-MCAO and EX-MCAO-EXO groups was not continuous, but it was more numerous, denser, and longer. Among them, the parameters of the EX-MCAO-EXO group were better than the EX-MCAO group.

### Neurological Function and Gait Analyses

**Figure 7:**
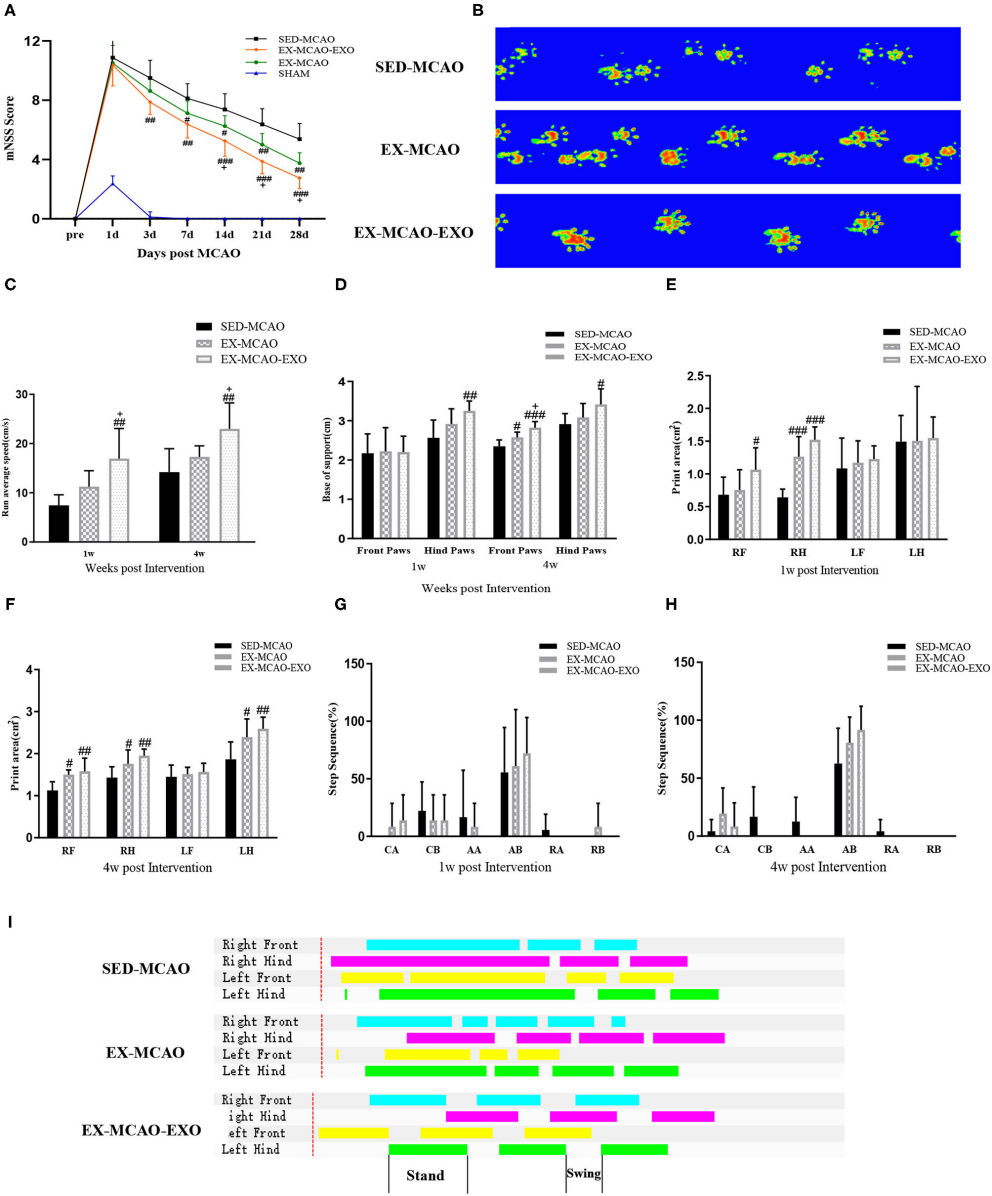
Neurological results and gait analysis of rats after the exercise intervention. **(A)** mNSS score. Means ± standard deviations, *n* = 8/group. **(B)** Representative paw prints. **(C)** The average movement speed of MCAO rats after 1 w and 4 w of exercise intervention. **(D)** The base of support of MCAO rats after 1 w and 4 w of exercise intervention. **(E,F)** The paw print area of the four paws of MCAO rats after 1 w and 4 w of exercise intervention. **(G,H)** Step sequence of MCAO rats after 1 w and 4 w of exercise intervention. **(I)** The plain view of the gait. LF, left forepaw; RF, right forepaw; LH, left hindpaw; RH, right hindpaw (R is the affected side). Step sequence: CA, right forepaw → left forepaw → right hindpaw → left hindpaw; CB, left forepaw → right forepaw → left hindpaw → right hindpaw; RA, right forepaw → left forepaw → left hindpaw → right hindpaw; RB, left forepaw → right forepaw → right hindpaw → left hindpaw; AA, right forepaw → right hindpaw → left forepaw → left hindpaw; AB, left forepaw → right hindpaw → right forepaw → left hindpaw. ^#^*p* < 0.05, ^##^*p* < 0.01, and ^###^*p* < 0.001 compared with the corresponding SED-MCAO group. ^+^*p* < 0.05 compared with the corresponding EX-MCAO group. Means ± standard deviations, *n* = 6/group.

The mNSS score was recorded before and 3, 7, 14, 21, and 28 days after the intervention. Before the intervention, no differences in the mNSS score were observed among the SED-MCAO, EX-MCAO, and EX-MCAO-EXO groups (Figure 7A), indicating that none of rats in the three groups showed significant differences in the degree of nerve deficits before treatment. Evaluated by repeated measures analysis of variance, mNSS scores were affected by time and intervention [*F*_time (5,105)_ = 202.456, *p*_time_ = 0.000; *F*_intervention(2,21)_ = 17.032, *p*_intervention_ = 0.000]. There was no interaction between time and intervention on mNSS score [*F*_time×*intervention*(10,105)_ = 1.763, *p* = 0.077]. A downward trend was found in three groups. Compared with the SED-MCAO group, the EX-MCAO-EXO group had a significantly decreased mNSS score from 3 days until the end of the experiment (*p*_3d_ = 0.004, *p*_7d_ = 0.001, *p*_14d_ = 0.000, *p*_21d_ = 0.000, *p*_28d_ = 0.000). The EX-MCAO group had a significantly decreased mNSS score from 7 days until the end of the experiment (*p*_7d_ = 0.041, *p*_14d_ = 0.027, *p*_21d_ = 0.006, *p*_28d_ = 0.001). The mNSS score of the EX-MCAO-EXO group was significantly lower than that of the EX-MCAO group beginning on the 14th day (*p*_14d_ = 0.047, *p*_21d_ = 0.020, *p*_28d_ = 0.027), which indicated that the EXO infusion enhanced the exercise effects.

We analyzed the gait using the CatWalk gait analysis system. Figure 7B shows the representative paw prints of each group. Paw prints in both the EX-MCAO and EX-MCAO-EXO groups were darker than those in the SED-MCAO group. The average movement speed in all groups showed an increasing trend (Figure 7C). After 1 week of intervention, the average speed of the EX-MCAO-EXO group was higher than that of the EX-MCAO group (*p*_1w_ = 0.022, p_4w_ = 0.037), but a significant difference was not observed between the SED-MCAO and EX-MCAO groups (*p*_1w_ = 0.137, *p*_4w_ = 0.231). The EX-MCAO-EXO group (*p*_1w_ = 0.022, *p*_4w_ = 0.037) had a significantly higher average speed than the EX-MCAO group.

The base of support (BOS) is the average width between either the forepaw or the hindpaw. After 1 week of intervention, the hindpaw BOS in the EX-MCAO-EXO group was higher than that in the SED-MCAO group (*p* = 0.006) (Figure 7D). After 4 weeks of intervention, the EX-MCAO-EXO group exhibited a significant increase in the forepaw BOS compared with the EX-MCAO group (*p* = 0.012), while EX-MCAO group displayed a significant increase in the forepaw BOS compared with the SED-MCAO group (*p* = 0.018).

After the 1 week intervention, the right forelimb paw print area in the EX-MCAO-EXO group (*p* = 0.045) was larger than that in the SED-MCAO group (Figure 7E). The right hind limb paw print area in the EX-MCAO group (*p*_EX−MCAO−RH_ = 0.000) and EX-MCAO-EXO group (*p*_EX−MCAO−EXO−RH_ = 0.000) was significantly higher than that in the SED-MCAO group. After 4 weeks intervention (Figure 7F), compared with the SED-MCAO group, the paw print area of the right forelimb, right hind limb, and left hind limb of the EX-MCAO group and the EX-MCAO-EXO group were significantly larger (*p*_EX−MCAO−RF_ = 0.011, *p*_EX−MCAO−RH_ = 0.045, *p*_EX−MCAO−LH_ = 0.03; *p*_EX−MCAO−EXO−RF_ = 0.003, *p*_EX−MCAO−EXO−RH_ = 0.003, *p*_EX−MCAO−EXO−LH_ = 0.005).

The step sequence of rats is mainly the AB type. After 4 weeks of intervention, the proportion of AB-type gaits increased and the ratio of AA and RB in unstable gaits decreased in the MCAO groups (Figures 7G,H). Figure 7I shows the plain view of the gait of each group. The standing phase time of the EX-MCAO and EX-MCAO-EXO groups was shorter, and the standing phase and swinging phase alternated more regularly.

### Correlation Analysis of Brain Exosome Levels With the mNSS Score, Infarct Volume Ratio, rFA, rADC, Syn, and PSD-95 Protein Expression Levels

**Figure 8:**
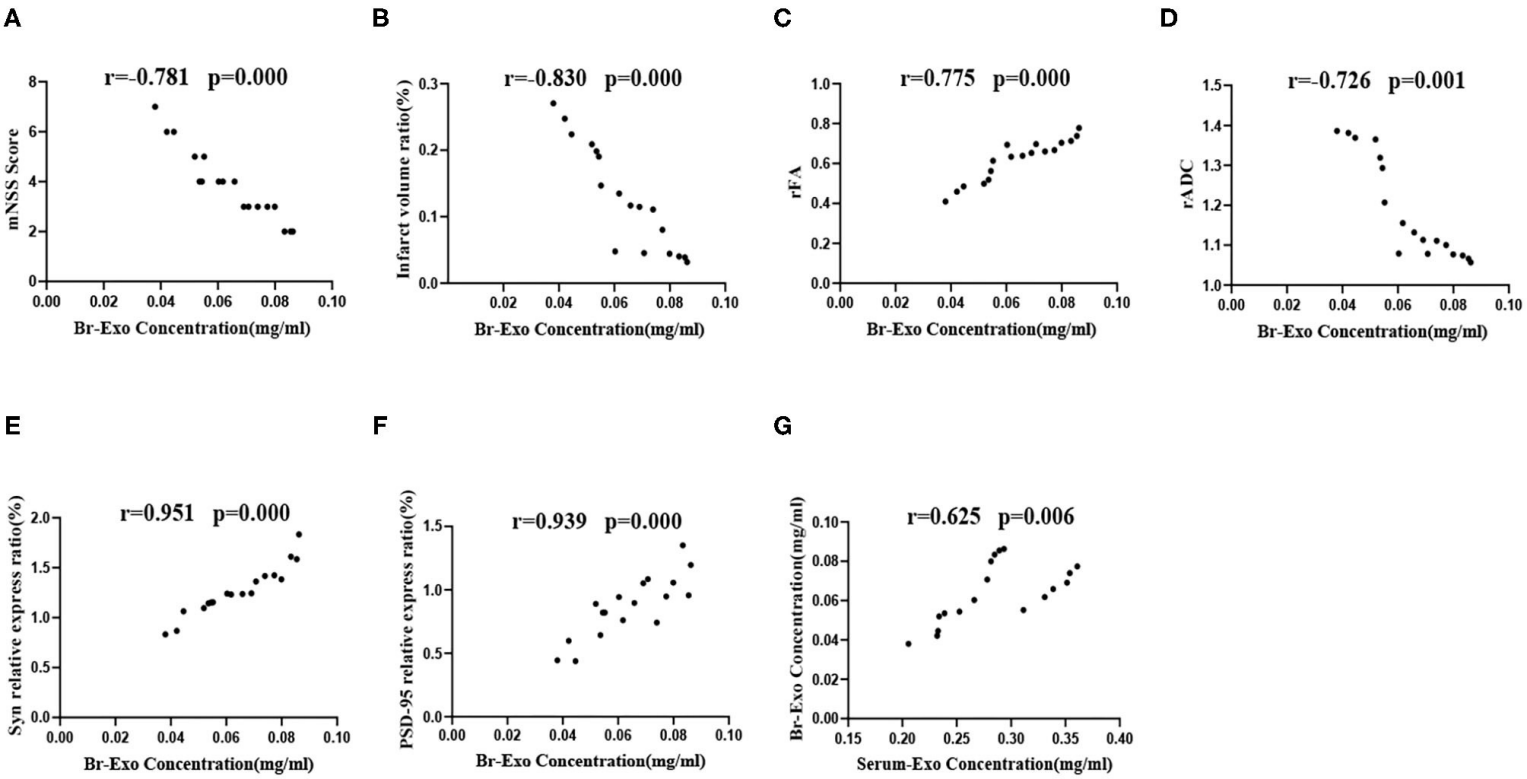
Correlation analysis of brain exosome levels with the mNSS score, infarct volume ratio, rFA value, rADC value, Syn, and PSD-95 protein expression levels. **(A–F)** Correlation analysis between brain exosome levels and the mNSS score, infarct volume ratio, rFA value, rADC value, Syn, and PSD-95 protein expression level. **(G)** Correlation analysis between serum exosome and brain exosome concentrations. Serum-Exo concentration: serum exosome concentration, Br-Exo concentration: brain exosome concentration. Means ± standard deviations, *n* = 6/group.

After 4 weeks of intervention, brain exosomes were extracted from the SED-MCAO, EX-MCAO, and EX-MCAO-EXO groups. According to the Pearson correlation analysis, the brain exosome concentration (Figures 8A–F) was negatively correlated with the mNSS score (*r* = −0.781, *p* = 0.000), infarct volume ratio (*r* = −0.830, *p* = 0.000), and rADC value (*r* = −0.726, *p* = 0.001) and showed a positive correlation with the rFA value (*r* = 0.775, *p* = 0.000), Syn expression (*r* = 0.951, *p* = 0.000), and PSD-95 expression (*r* = 0.939, *p* = 0.000). The serum exosome concentration displayed a positive correlation with the brain exosome concentration (Figure 8G) (*r* = 0.625, *p* = 0.006).

## Discussion

This study reported several important findings. (1) After 1 week of intervention, both the EX-MCAO and EX-MCAO-EXO groups had significantly higher serum exosome levels than the SED-MCAO group. After 4 weeks of intervention, both the EX-MCAO and EX-MCAO-EXO groups showed higher serum exosome levels than the SED-MCAO group. The EX-MCAO-EXO groups had significantly lower serum exosome levels than the EX-MCAO group. (2) After 1 and 4 weeks of intervention, both the EX-MCAO and EX-MCAO-EXO groups displayed higher brain exosome levels than the SED-MCAO groups. The EX-MCAO-EXO group had the highest brain exosome levels among the three groups. (3) After 4 weeks of intervention, compared with the SED-MCAO group, the EX-MCAO and EX-MCAO-EXO groups had significantly improved synapse numbers, synaptic plasticity-associated protein expression, infarct volume ratio, rFA and rADC, mNSS score, and gait ability. The EX-MCAO-EXO group displayed a greater improvement than the EX-MCAO group. (4) Pearson's correlation analysis revealed that the brain exosome levels were negatively correlated with the mNSS score, infarct volume ratio, and rADC value and positively correlated with the rFA value, Syn, and PSD-95 expression. In addition, serum exosome levels were positively correlated with brain exosome levels. Our study indicated that MCAO rats that received the exercise intervention increased serum and brain exosome levels and synaptic plasticity-associated protein expression, the induction of neuroplasticity and enhanced motor recovery, and that an exosome injection could enhance the effects.

### Exercise Intervention Increases the Serum and Brain Exosome Levels in MCAO Rats, and the Ischemic Brain Shows Strong Attraction for Exosomes

According to some studies, exercise intervention promotes the release of exosomes into the circulation (42, 43). Circulating exosomes are derived from a variety of cell types, such as neurons, dendritic cells, and mesenchymal cells (44), and those exosomes can cross the blood-brain and blood-cerebrospinal fluid barriers under normal and disease conditions to communicate between the periphery and the brain (45). Hou et al. found that plasma exosomes isolated from exercise rats provide significant protection against myocardial ischemia/reperfusion injury (46). Indium 111-labeled exosomes injected through the tail vein 1 h after MCAO accumulate in the peri-infarcted area of the brain (12) and then promote white matter remodeling, angiogenesis, and neurogenesis (16), indicating that exosomes cross the BBB and participate in the brain's response to ischemia (47, 48). *In vivo* imaging demonstrated that DIR-labeled exosomes can cross the blood-brain barrier from circulation into the brain.

As shown in the present study, MCAO rats had increased serum and brain exosome levels, which may be due to tissue damage. Furthermore, exercise intervention increased serum and brain exosome levels in MCAO rats. Wang et al. found that exercise increases plasma and brain tissue levels of circulating endothelial progenitor cell-derived exosomes (15), suggesting that exercise may increase exosome levels in brain tissue and have a long-term beneficial effect on MCAO.

Interestingly, after 4 weeks of exercise training, the serum exosome level in the EX-MCAO group was higher than that in the EX-MCAO-EXO group, while the brain exosome level in the EX-MCAO group was lower than that in the EX-MCAO-EXO group. This discrepancy may be because the ischemic brain attracts exosomes from the circulation and promotes their transport across the BBB. Exosomes then react with receptors on brain cells to promote the release of more exosomes, which results in cell-to-cell communication and promotes neural function recovery (16). Studies have confirmed that circulating exosomes are the source of brain exosomes (15), but it cannot be ruled out that exercise can directly increase the level of exosomes in the brain.

### Exercise Intervention Increases the Number of Synapses and Improves Synaptic Plasticity

Cerebral infarction leads to nerve cell damage and death and reduces the number of synapses, which is important for neurological dysfunction (49). Exercise intervention increases the synapse number and synaptic plasticity-associated protein expression in the rat brain (5, 50, 51). Neuronal exosomes are present at synaptic level, and exosomal signals have been shown to affect synaptic plasticity, neuroprotection, etc. Studies have shown that exosomes isolated from miR-133b-overexpressing mesenchymal stem cells significantly increase the functional improvement and brain plasticity (45). Li et al. reported that exosomes derived from neural stem cells increase the Syn expression level (52). Our study found that exercise intervention increased the number of synapses, Syn, and PSD-95 expression in the brains of MCAO rats. After exosome injection, the synapse number, Syn, and PSD-95 expression were further increased, which revealed that exosome injection had a superimposed effect on synaptic plasticity.

### Exercise Intervention Reduces the Infarct Volume and Improves CST Integrity

MRI scans assess the severity of cerebral infarction (22), and DTI detects the integrity of the CST *in vivo*. A decrease in the FA value and an increase in the ADC value indicate that nerve fibers are damaged (23, 26). Reconstruction of the CST by DTT technology enables researchers to further observe the damage and predict the outcome of exercise. Our previous experiments confirmed that starting an exercise intervention early after stroke (1 day after MCAO) reduces the infarct volume ratio in rats and enhances neuroplasticity (34, 53). Intravenously injected exosomes derived from mesenchymal stem cells were found to enhance neurite remodeling (17) and fiber bundle integrity (39) and induce significant axial bud response (17). As shown in studies by Zheng et al., exosomes reduce the cerebral infarct volume in rats with cerebral ischemia within 6 h of reperfusion (54) and exert neuroprotective effects (55). In the present study, the exercise intervention reduced the infarct volume and enhanced the CST of the affected side. Furthermore, the exercise intervention and exosome infusion showed additional benefits in reducing the area of cerebral infarction and improving the intact CST on the affected side. Our results suggested that exercise intervention regulated exosomes and ameliorated brain and CST damage in rats with MCAO.

### Exercise Intervention Improves Neurological Function and Gait Ability

The mNSS score is a comprehensive motor, sensory, balance, and reflex score commonly used after MCAO (56), and the change in the mNSS score reflects the improvement or deterioration of neurological function. Exercise intervention has been reported to reduce the mNSS score (57). In the present study, we did not observe difference in mNSS scores 1 day after MCAO among the SED-MCAO, EX-MCAO, and EX-MCAO-EXO groups, indicating the lack of a significant difference in the severity of the functional deficits among the three groups before the intervention. The EX-MCAO and EX-MCAO-EXO groups had significantly reduced mNSS scores compared with the SED-MCAO group. The score of the EX-MCAO group decreased at 7 days and the score of the EX-MCAO-EXO group decreased at 3 days after exercise intervention, and the decreasing trends continued for 4 weeks. The mNSS score of the EX-MCAO-EXO group was significantly lower than that of the EX-MCAO group beginning at 14 days after intervention. The results indicated that the exercise intervention improves neurological function and that exosome input exerts an additive effect on improving neurological function.

The CatWalk gait analysis system is considered an ideal tool for rat neurobehavioral testing (58) and a more stable and reliable non-subjective prognostic indicator. It can detect long-term sensorimotor deficits after MCAO and reveal the relationship between behavioral deficits and ischemia (59).

In this study, after the exercise intervention, MCAO rats exhibited an increased average speed and affected paw print area, indicating that exercise intervention increased the strength and weight-bearing function of the affected limb. The base of support (BOS) and the step sequence are indicators that reflect coordination (60). The BOS is the average width between either the forepaw or the hindpaw, and an increased BOS indicates that unstable gait is compensated (59). This study indicated an increase in the BOS after the exercise intervention, revealing that the exercise intervention stabilized gait. In healthy rats, the AB pattern (left forepaw → right hindpaw → right forepaw → left hindpaw) was the most common step sequence (61). After the exercise intervention, the proportion of the most common steps of the AB type gait increased, and the ratio of unstable rotation AA and RB type steps decreased, indicating an increase in the coordination of the rat. The aforementioned results show that exercise intervention improves gait parameters and promotes the recovery of motor function and overall balance and coordination of rats with cerebral ischemia.

### The Level of Brain Exosomes Is Correlated With Synaptic Growth, CST Integrity, the Infarct Volume, and Motor Function

Single doses of exosomes can upregulate genes related to neurogenesis, neurotransmission, and neuroplasticity, resulting in neuroprotective effects (62). Moderate exercises can regulate the secretion of circulating endothelial progenitor cell-derived exosomes, which are the source of endothelial progenitor cell-derived exosomes, and contribute to the recovery of brain function (15). In the present study, we analyzed the correlations between the brain exosome levels and the mNSS score, infarct volume ratio, rFA value, rADC value, Syn, and PSD-95 protein expression level. Furthermore, we analyzed the relationship between serum exosome and brain exosome concentrations. We found that brain exosome levels were associated with neurological recovery (mNSS, MRI parameters) and were highly correlated with synaptic plasticity-associated proteins. Serum exosome levels were positively correlated with brain exosome levels. These results suggest that exercise intervention has the ability to increase the level of circulating exosomes, which can cross the blood-brain barrier and target into the ischemic brain, thereby increasing the level of exosomes in the ischemic brain. These exosomes regulate synaptic plasticity, improve corticospinal tract integrity, and produce neuroprotective effects that enhance motor performance.

#### Advantage of the Study

In this study, brain-derived exosomes were selected for injection, because it cannot be ruled out that exosomes derived from specific cells induce the release of other neural parenchymal cells to preferentially enhance neuroplasticity (63). In addition, exosomes derived from *in vitro* cannot provide information on the *in vivo* physiological effects of exosomes secreted by multiple cell types under normal, developmental, and pathological conditions (64). However, exosomes isolated directly from brain tissue can reveal the processes of the normal brain and the changes in the brain environment under disease conditions *in vivo* (64). A study revealed that in myocardial infarction models, human induced pluripotent stem cell-derived exosomes extracted from a mixture of cardiomyocytes, smooth muscle cells, and endothelial cells had better efficacy than these three cells alone (65).

Currently, studies have assessed neuroprotection induced by exercise alone and exosome infusion therapy, exosomes produced by exercise, and the involvement of exosomes in brain function repair. All these studies are independent. Research on the effects, relationship, and mechanism of exercise intervention on exosomes and neuroplasticity after cerebral ischemia is rare. In the present study, we clarify that changes in exosome levels may be the mechanism by which exercise intervention promotes neuroplasticity. This research is expected to provide new treatment strategies for the clinic.

#### Limitations of the Study

In the present study, MRI and gait analyses were performed only 1 and 4 weeks after the exercise intervention, and no additional time points were detected. Exosomes from different sources contain different types of miRNA, proteins, etc., but the types of substances contained in exosomes have not been analyzed yet. Another limitation is that we did not carry out experiments with exosome inhibitors combined with exercise intervention in MCAO rats. In addition, we cannot rule out the possibility that exercise can directly induce the biogenesis of exosomes in the brain. These limitations will be addressed in future experiments.

## Summary

Exercise intervention promotes synaptic plasticity-associated protein expression by adjusting the content of exosomes, improves neuroplasticity, and thus improves exercise capacity. This study may provide a new target for the rehabilitation of stroke and a new insight to improve the efficacy of rehabilitation.

## Data Availability Statement

The raw data supporting the conclusions of this article will be made available by the authors, without undue reservation.

## Ethics Statement

The animal study was reviewed and approved by the Ethics Committee of Tianjin Medical University.

## Author Contributions

All authors listed have made a substantial, direct and intellectual contribution to the work, and approved it for publication.

## Funding

This work was supported by the National Key R&D Program of China [grant numbers 2017YFC1308504 and 2017YFC1104004], Tianjin Special Branch Plan High-level Innovation Team Grant, Natural Science Foundation of Tianjin, China [grant number 18JCZDJC98900], and Tianjin Key Medical Discipline Construction Project.

## Conflict of Interest

The authors declare that the research was conducted in the absence of any commercial or financial relationships that could be construed as a potential conflict of interest.

## Publisher's Note

All claims expressed in this article are solely those of the authors and do not necessarily represent those of their affiliated organizations, or those of the publisher, the editors and the reviewers. Any product that may be evaluated in this article, or claim that may be made by its manufacturer, is not guaranteed or endorsed by the publisher.
