# Supplementary material for: Exercise Intervention Promotes the Growth of Synapses and Regulates Neuroplasticity in Rats With Ischemic Stroke Through Exosomes
Source: Front Neurol. 2021 Oct 28;12:752595. doi: 10.3389/fneur.2021.752595 (PMC8581302; doi:10.3389/fneur.2021.752595)

The MCAO model

1. After the rats awakened, a Longa score of 1-3 points was used as the standard for successful modeling.

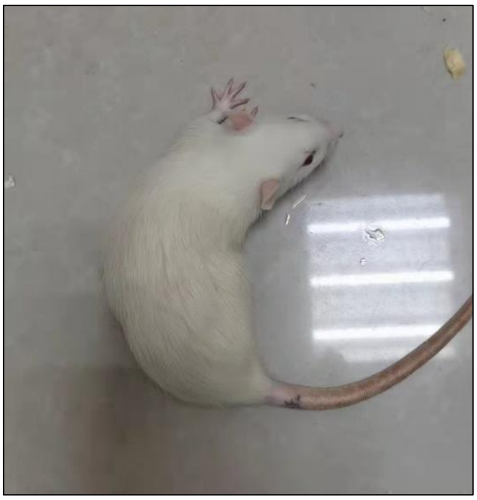
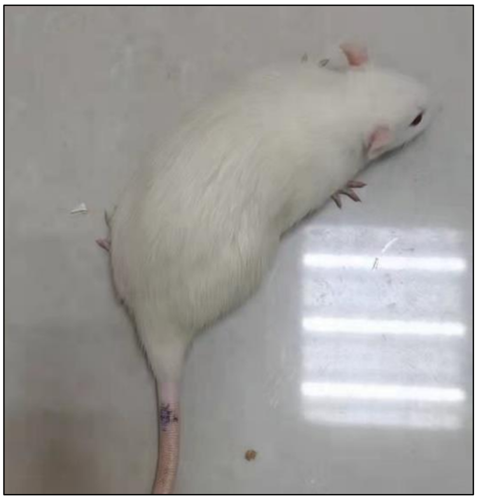
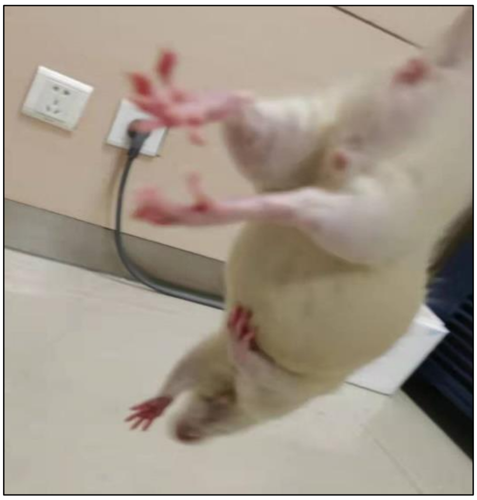
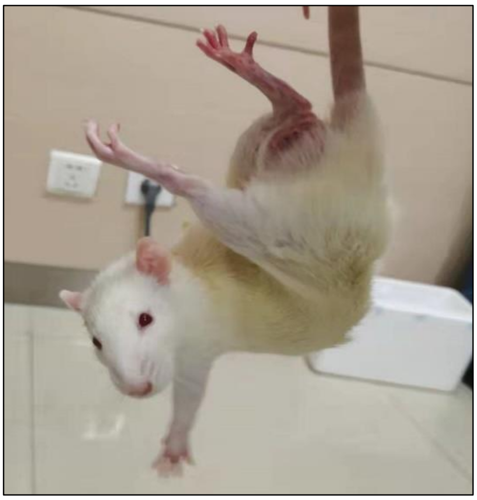

2. TTC staining 1d after MCAO. Statistical analysis showed that there was no difference in the infarct volume ratio.

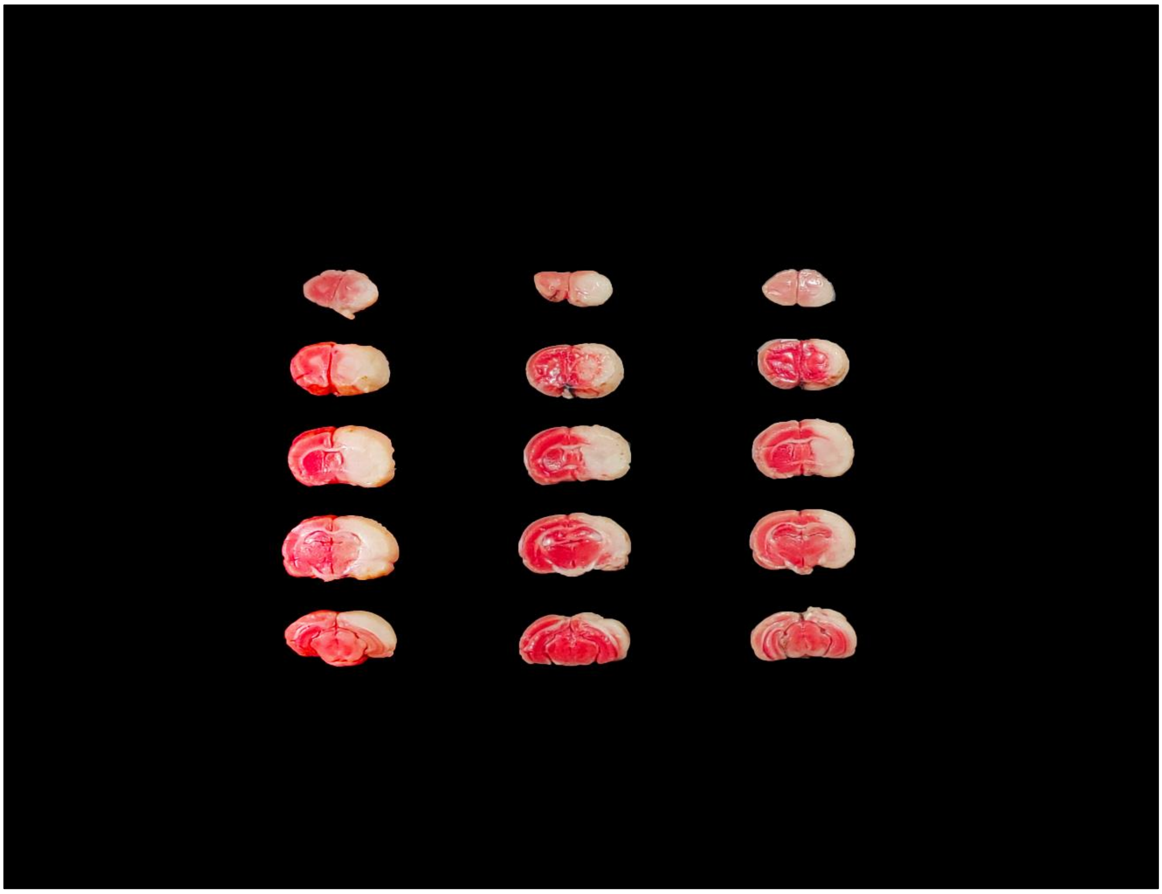

Supplement: Supplementary file 1 [file Data_Sheet_1.PDF]
